# Autosomal dominant polycystic kidney disease: an overview of recent genetic and clinical advances

**DOI:** 10.1080/0886022X.2025.2492374

**Published:** 2025-04-23

**Authors:** Abdul Hamid Borghol, Marie Therese Bou Antoun, Christian Hanna, Mahdi Salih, Frederic F. Rahbari-Oskoui, Fouad T. Chebib

**Affiliations:** ^a^ Division of Nephrology and Hypertension, Department of Medicine, Mayo Clinic, Jacksonville, FL, USA; ^b^ Mayo Clinic Florida PKD Center of Excellence, Jacksonville, FL, USA; ^c^ Division of Pediatric Nephrology and Hypertension, Department of Pediatric and Adolescent Medicine, Mayo Clinic, Rochester, MN, USA; ^d^ Division of Nephrology and Hypertension, Department of Medicine, Mayo Clinic, Rochester, MN, USA; ^e^ Division of Nephrology and Transplantation, Department of Internal Medicine, Erasmus Medical Center, Rotterdam, The Netherlands; ^f^ Renal Division, Department of Medicine, Emory University School of Medicine, Atlanta, GA, USA

**Keywords:** ADPKD, polycystic kidney disease, Polycystic liver disease, tolvaptan, total kidney volume

## Abstract

Autosomal Dominant Polycystic Kidney Disease (ADPKD) is the most common inherited kidney disease, characterized by the progressive development of multiple kidney cysts, leading to a gradual decline in kidney function. ADPKD is also the fourth leading cause of kidney failure (KF) in adults. In addition to kidney manifestations, ADPKD is associated with various extrarenal features, including liver cysts, cardiovascular abnormalities, intracranial aneurysms, and chronic pain with significant impact on patients’ quality of life. While several disease-modifying agents have been tested in ADPKD, tolvaptan remains the only approved drug by the US Food and Drug Administration. The Mayo Imaging Classification is currently the most practical tool for predicting rate of kidney disease progression in ADPKD. This review provides a comprehensive overview of ADPKD, focusing on its genetics, pathophysiology, clinical presentation, management, and prognostic tools. Advances in diagnostic imaging and genetic testing have improved the early detection of ADPKD, allowing better classification of patients and prediction of KF. The review also discusses current therapeutic approaches to ADPKD, including tolvaptan, a vasopressin V2-receptor antagonist. Additionally, we address specific issues in children and pregnant individuals with ADPKD. Despite substantial progress in understanding ADPKD, there is a large need for additional effective treatments and prognostic markers to provide a more personalized care for these patients.

## Introduction

Autosomal Dominant Polycystic Kidney Disease (ADPKD) is the most common genetic kidney disorder, predominantly caused by pathogenic variants in the *PKD1* and *PKD2* genes [1]. Recently, additional pathogenic variants in other genes have been identified, broadening the genetic landscape of ADPKD [2]. The disease is marked by the progressive development of kidney cysts, leading to kidney enlargement and eventual kidney failure (KF). ADPKD accounts for 5–10% of all KF cases, making it the fourth cause of KF after diabetes mellitus, hypertension, and glomerulonephritis [3,4]. Beyond kidney involvement, ADPKD is a systemic disease with significant extrarenal manifestations, including liver and pancreatic cysts, valvular heart disease, and an increased risk of intracranial aneurysms [1,4]. Despite extensive research into therapeutic options, tolvaptan, a V2-receptor antagonist, remains the only United States (U.S.) Food and drug Administration (FDA)-approved disease-modifying treatment for patients with a high risk of rapidly progressing kidney disease [5]. This review explores the multifaceted aspects of ADPKD, including its epidemiology, etiology, genetics, diagnostic criteria, clinical manifestations, management strategies, and prognosis.

## Epidemiology

ADPKD affects up to 12 million people worldwide, with an estimated annual incidence of 2.5 cases per 100,000 individuals [1,4,6]. The prevalence of ADPKD has been assessed using various methods, including population-based imaging studies, genetic testing for pathogenic variants, and clinical diagnostic criteria [7]. The disease is observed across all races and ethnicities, although its prevalence varies. Higher rates are reported in Black individuals (73 per 100,000) and non-Hispanic White individuals (63.2 per 100,000), compared to Asian/Pacific Islanders (48.9 per 100,000) and Hispanics (39.9 per 100,000) [8].

Recent insights from large-scale genomic studies have refined the understanding of ADPKD prevalence. In these studies, ADPKD was restricted to cases secondary to pathogenic variants in the *PKD1* and *PKD2* genes (excluding minor PKD genes) [6]. Lanktree et al. examined two large whole genome sequencing (WGS) and whole exome sequencing (WES) databases (gnomAD and BRAVO) and estimated the likely true prevalence of ADPKD to be approximately ∼1 in 1072 [6]. This figure is higher than epidemiologic estimates (∼1 in 2000), highlighting the potential underestimation of milder or asymptomatic cases in large population studies [9–11].

In the United States, ADPKD accounts for approximately 5% of new dialysis cases annually. Rates of KF are notably higher in males than females (8.2 vs 6.8 cases per million population, respectively) [12]. Additionally, disparities in disease progression are evident; non-Hispanic Black individuals with ADPKD reach KF at a younger age compared to non-Hispanic White individuals (54.4 vs 55.9 years, *p* < 0.0001) [12,13]. Contributing factors may include comorbid conditions, such as sickle cell trait, and genetic risk factors, including *APOL1* risk alleles [13,14].

## Etiology and genetics

ADPKD is inherited in an autosomal dominant pattern, characterized by high penetrance but variable expression, with an equal sex distribution [15]. Disease presentation and severity often vary even among family members, reflecting the interplay of genetic, epigenetic, and environmental factors [4,15,16]. Studies reveal notable variability in ADPKD progression. Identical twins differ by an average of 2 years in kidney failure onset [17]. For siblings with the same PKD variant, the difference increases to 7 years, while family members overall exhibit an average difference of 13 years [17–19]. The majority of ADPKD cases result from pathogenic variants in two key genes: *PKD1* on chromosome 16 (accounting for ∼78% of cases) and *PKD2* on chromosome 4 (∼15%) [2,20,21]. Additionally, pathogenic variants in other genes, including *IFT140*, *DNAJB11*, *GANAB*, *NEK8*, and *ALG5*, *ALG8*, *ALG9* contribute to a smaller portion (<7%) of genetic diagnoses in ADPKD [20–25]. The Kidney Disease: Improving Global Outcomes (KDIGO) 2025 guidelines recommend a standardized nomenclature where the disease name is followed by the gene (e.g., ADPKD-*PKD1* or ADPKD-*PKD2*) to enhance clarity and alignment with classification standards [7]. These findings expand the genetic landscape of ADPKD and are summarized in Table 1 and Supplemental Figure S1.

**Table 1.** Comparison of the different genetic factors, renal and extrarenal manifestations and prognosis among patients with ADPKD.

Variant	Disease	Chr.	Protein	Protein function	Prop.	Renal Manifest.	Extrarenal manifest.	Prognosis
*PKD1*	ADPKD-*PKD1*	16p13.3	Polycystin-1	-Receptor, complex with PC2 to form polycystin Ca2+ channel on primary cilia -Tubulogenesis (not well understood)	78–85%	Multiple bilateral cysts. Highest cystic burden among other pathologic variants	Increased risk of IA (unclear Rate) Absent to severe PLD	Median age at onset of KF is 54-year-old (Range 53–60)
*PKD2*	ADPKD-*PKD2*	4q21	Polycystin-2	Ca2+-permeable nonselective cation channel. Complex with PC1	10–15%	Multiple bilateral cysts	-Increased risk of IA (Unclear rate) -Absent to severe PLD	Median age at onset of KF is 77.8 (Range 60 − 84.6)
*IFT140*	ADPKD-*IFT140*	16p13.3	IFT140 protein (Part of IFT complex-A)	-Retrograde ciliary transport. -development and functioning of cilia	1–2%	Few, large bilateral cysts, asymmetrical enlarged Kidneys	PLD rare	Preserved GFR until old age
*GANAB*	ADPKD-*GANAB*	11q12.3	α-Subunit of glucosidase-II	Catalytic subunit of glucosidase II. PC1&PC2 complex trafficking to the cilia and cell surface	>0.5%	Mild cystic burden	Mild to severe PLD	Limited CKD, no KF
*DNAJB11*	ADPKD-*DNAJB11*	3q27.3	DNAJ heat shock protein 40 subfamily B, member 11	Co-chaperone for HSPA5. Maturation and correct trafficking of PC1	>0.5%	-Bilateral, small cysts, mild enlargement -High incidence of nephrolithiasis	-Possible IA -Mild PLD -Diabetes	Limited early CKD, KF in 70s
*ALG5*	ADPKD-*ALG5*	13q13.3	Dolichyl-phosphate beta-glucosyl-transferase	Assembly of oligosaccharides in kidney epithelial cells. Proper PC1 glycosylation and maturation	<0.5%	-Mild-moderate cystic burden, mild enlargement -Possible nephrolithiasis	–	CKD, KF in older patients due to fibrosis
*ALG8*	ADPKD-*ALG8*	11q14.1	α-3-glucosyl-transferase	-Glycosylation of lipid-linked oligosaccharides -Maturation and localization of PC1 into the primary cilia	∼1%	-Mild cystic disease -Possible nephrolithiasis	Mild to severe PLD (ADPLD)	Preserved GFR into old age
*ALG9*	ADPKD-*ALG9*	11q23.1	α-1,2-mannosyl-transferase	-Transfer of mannose into lipid-linked oligosaccharides -Proper PC1 maturation	>0.5%	-Mild to moderate cystic disease -Possible nephrolithiasis	PLD is common	Significant CKD in older individuals
*PKHD1*	ADPKD-*PKHD1*	6p12.3–p12.2	Fibrocystin	-Ciliogenesis, Tubulogenesis. -Cell-Cell and Cell-ECM interactions.	∼1%	Generally, very mild cystic disease	PLD is common	Preserved function into old age.

ADPKD: autosomal dominant polycystic kidney disease, PC1: polycystin 1, PC2: polycystin 2, IA: intracranial aneurysm, PLD: polycystic liver disease, ADPLD: autosomal dominant polycystic liver disease, KF: Kidney failure, CKD: chronic kidney disease, GFR: glomerular filtration rate, HSPA5: heat shock protein family A member 5, ECM: extracellular matrix.

The *PKD1* gene encodes polycystin-1 (PC1), a multidomain membrane protein involved in extracellular interactions and intracellular signaling pathways that regulate cell proliferation [26]. PC1 is expressed in key structures implicated in cyst formation, such as kidney tubular epithelial cells, hepatic bile ducts, endothelial cells, and pancreatic ducts [27]. *PKD2* encodes polycystin-2 (PC2), a calcium-regulated cation channel of the transient receptor potential (TRP) family, found in kidney tubular epithelial cells, vasculature, hepatic cells, brain, and placenta [28,29]. Both PC1 and PC2 are localized to non-motile primary cilia (supplemental Figure S2), where their interaction is essential for PC1 stabilization, trafficking, and maturation [30,31].

A key challenge in the genetic diagnosis of ADPKD involves the presence of *PKD1* pseudogenes. The *PKD1* locus is flanked by six highly homologous pseudogenes on chromosome 16, which share ∼97.7% sequence similarity with the 5′ region of *PKD1*. These pseudogenes complicate the accurate detection of pathogenic variants in *PKD1*, necessitating the use of advanced sequencing techniques, such as long-read sequencing or locus-specific capture, to distinguish between functional *PKD1* and its pseudogenes [32,33].

Other genes implicated in ADPKD affect PC1 trafficking or glycosylation in the endoplasmic reticulum (ER), including *GANAB*, *PRKCSH*, *ALG8*, *PMM2*, *SEC63*, *SEC61A1*, and *SEC61B* [4,16,34]. Mosaicism, a condition characterized by the presence of distinct cell lineages due to *de novo* mutations occurring during embryogenesis, has also been reported in ADPKD [35]. The clinical variability in such cases depends on the type of cells affected and the timing of the mutation during development [16]. While *PKD1* and *PKD2* variants are the primary causes of ADPKD, other cystic kidney disease-associated genes can either contribute to similar cystic diseases or influence ADPKD onset and severity [15]. *PKHD1* and *HNF1B*, key regulators of ciliary protein genes, impact ADPKD progression, with *HNF1B* being a transcription factor that can upregulate the expression of multiple PKD-associated genes (e.g., *PKHD1* and *PKD2*) [15,36]. Biallelic *PKHD1* mutations cause autosomal recessive polycystic kidney disease (ARPKD) while monoallelic variants cause ADPKD with a milder presentation [2,15]. *HNF1B* variants, associated with autosomal dominant tubulointerstitial disease (ADTKD), lead to *HNF1B-*related nephropathy [37]. TSC genes are also important: *TSC1* and *TSC2* mutations are implicated in tuberous sclerosis complex (TSC), presenting with simple cysts and renal angiomyolipomas, or in *TSC2*/*PKD1* contiguous gene deletion syndrome, leading to early-onset cystic disease [38,39]. *TSC* mutations disrupts the mTOR pathway, accelerating cyst growth and ADPKD progression (Supplemental Figure S3). Furthermore, compound heterozygosity, where a *PKD1* variant coexists with another cystic gene mutation (e.g., *PKD2*, *COL4A1*, or *HNF1B*), has been linked to worsened disease severity [40–42]. Approximately 10% of clinically detected ADPKD cases test negative for *PKD1* and *PKD2* variants. This may be due to ADPKD genes that are newly discovered, genes that are not yet discovered, or to deep intronic variations affecting gene splicing, which necessitates comprehensive sequencing of both coding and non-coding regions for accurate genetic diagnosis [43,44].

## Pathophysiology

The polycystin complex (PC), composed of PC1 and three PC2 proteins, functions as a heteromeric cation channel complex [45]. This complex localizes to the primary cilia, a signaling hub on the apical surface of tubular epithelial cells [46,47]. Structural studies (Supplementary Figure S2) demonstrate that PC1 and PC2 assemble in a 1:3 ratio, forming a TRP-like ion channel complex essential for calcium homeostasis [48]. Genetic variants in *PKD1* and *PKD2* can disrupt this structure at multiple levels. Truncating *PKD1* variants prevent proper assembly of the PC1-PC2 complex, leading to early-onset disease and more severe cystogenesis [45]. Missense mutations affecting key structural domains, such as the extracellular TOP domain, PLAT domain, and terminal domains impair PC1-PC2 interactions, disrupting calcium transport and increasing cAMP signaling [45,48]. Missense mutations in *PKD2*, particularly at Asp511 in the voltage-sensing domain (VSD), disrupt conformational changes in S1-S4, altering ion selectivity, permeability, and calcium conductance. These defects impair ciliary signaling, leading to dysregulated calcium homeostasis and enhanced cystic expansion in ADPKD [26,49].

The polycystin complex plays a central role in regulating intracellular calcium levels, cyclic adenosine monophosphate (cAMP) signaling, and cellular homeostasis [50,51]. Pathogenic variants in PKD genes disrupt these pathways, reducing intracellular calcium, increasing cAMP levels, and activating protein kinase A (PKA) [50–53]. These changes drive cellular proliferation, fluid secretion, interstitial inflammation, and fibrosis, which collectively lead to cyst formation, destruction of non-cystic kidney parenchyma, and eventual KF [54–57]. While haploinsufficiency alone is sufficient for cyst formation in some cases, other cases may require a second somatic ‘hit’ to the normal allele, supporting the two-hit model of cystogenesis [58–60].

Arginine vasopressin (AVP) plays a pivotal role in the pathogenesis of ADPKD through its action on V2 receptors (V2R) on the basolateral membrane of renal tubular epithelial cells [61,62]. AVP binding to V2R activates adenylate cyclase, leading to increased cAMP levels, which are critical for cystogenesis [62–64]. Elevated cAMP activates PKA, exacerbating cystic expansion by promoting CFTR-mediated chloride-driven fluid secretion into the cyst lumen and increasing epithelial cell proliferation [55,65]. This amplification of cAMP signaling is shown schematically in supplemental Figure S3.

Minor PKD genes, such as *GANAB* and *PRKCSH*, play crucial roles in the maturation of PC1 within the endoplasmic reticulum and its subsequent impaired trafficking to the primary cilia, processes that are pivotal in the development of cysts [4,31,66]. Additionally, enlarging cysts compress renal vasculature, leading to ischemia and chronic activation of the renin-angiotensin-aldosterone system (RAAS), which underlies the early onset of hypertension in ADPKD. Hypertension, in turn, is an independent risk factor for accelerated kidney function decline [67].

Metabolic dysfunction is another hallmark of ADPKD [68]. Cystic cells exhibit a shift from oxidative phosphorylation to glycolysis, a phenomenon associated with mitochondrial dysfunction [69]. These findings have spurred interest in dietary interventions, including ketogenic diets and ketosis-inducing therapies [70]. Notably, experimental studies show that re-expression of polycystins in cystic kidneys can reverse the cystic phenotype, highlighting the therapeutic potential of targeting polycystin pathways [71].

## Diagnosis

ADPKD is primarily diagnosed through abdominal imaging, which typically reveals bilateral kidney cysts and kidney enlargement. Genetic testing is particularly valuable in specific scenarios, such as atypical presentations or when confirmation is required for family planning, risk stratification, or young potential kidney donors [1,72,73]. Accurate diagnosis and follow-up are best managed by a multidisciplinary team that includes pediatric and adult nephrologists, geneticists, and radiologists experienced in ADPKD [74]. Comprehensive counseling is essential to inform patients and families about the potential benefits and challenges associated with screening and genetic testing [73–75]. Clinicians should test for ADPKD in individuals with a family history of CKD, KF, PKD, or kidney cysts as well as in those with personal histories of CKD, hypertension, liver cysts, gross hematuria, or kidney stones [1,76].

Kidney US is the preferred method for screening symptomatic or presymptomatic individuals at risk of ADPKD due to its availability, affordability, and lack of radiation exposure [76]. Magnetic resonance imaging (MRI) and computed tomography (CT) scan are commonly used to confirm ADPKD diagnosis, measure height-adjusted total kidney volume (ht-TKV), and assess the risk of disease progression, and determine the need for Tolvaptan therapy [77–79]. The modified Pei-Ravine criteria for diagnosing ADPKD vary depending on age and the presence or absence of family history of the disease. Contrast-enhanced CT (CCT) and MRI, with their higher sensitivity for detecting small cysts, provide distinct diagnostic thresholds compared to US (Supplemental Table S1) [80]. In individuals over 40, the absence of cysts on US effectively rules out ADPKD, regardless of family history or known PKD variant [72]. For those under 40, MRI or CCT is preferred, as the presence of four or fewer cysts is sufficient to exclude the diagnosis in individuals with a family history of ADPKD [80]. Non-contrast MRI is sufficient to provide most of the necessary diagnostic information. However, in cases where incidental masses or complex cysts are detected, contrast-enhanced MRI with gadolinium is recommended for further evaluation [80,81]. Liver cysts, which are present in over 85% of individuals with ADPKD by the age of 30, can support the suspicion for a cystic disease, particularly in patients without family history of ADPKD [77,78]. Both CCT and MRI are reliable for calculating TKV, differentiating cystic from non-cystic tissue, and assessing cyst burden [1,82]. T2-weighted MRI is effective for visualizing kidney cysts, which appear hyperintense on T2 images, though its higher cost limits routine use [83]. Non-contrast CT is effective for detecting kidney stones but involves significant ionizing radiation, making frequent imaging impractical. Additionally, the use of iodinated contrast enhancement is typically avoided in moderate to advanced CKD [84]. Because of these risks, MRI is generally preferred over CCT for longitudinal monitoring, though MRI is unsuitable for detecting kidney stones or nephrocalcinosis [1,15,85].

### Genetic testing in ADPKD

Genetic testing is crucial when a diagnosis is unclear, particularly in atypical cases, absence of family history, early or very early ADPKD (diagnosed before age 15 or perinatally up to 18 months), KF without significant kidney enlargement, or when there is a discrepancy between eGFR decline and kidney cyst burden [42,74, 86,87]. Testing is also essential in scenarios such as preimplantation genetic diagnosis or assessing potential young living kidney donors at risk of ADPKD [88]. Additionally, genetic testing is recommended when extrarenal manifestations suggestive of syndromes other than ADPKD, such as ADTKD, tuberous sclerosis or Von Hippel-Lindau [87]. Recent advancements in next generation sequencing (NGS) allow for the simultaneous testing of multiple genes [41]. For instance, genetic testing has identified rare conditions such as the contiguous *TSC2/PKD1* deletion syndrome or a compound heterozygosity involving a truncating and a nontruncating *PKD1* variants [40,89]. Moreover, novel gene variants, including *DNAJB11*, *ALG9*, *COL4A3*, and *COL4A4* have been identified in patients with atypical or unusual imaging findings and no family history [15,42,58].

### Kidney disease progression and risk stratification

Several markers have been utilized to predict the disease course in ADPKD [90].

#### Serum creatinine and eGFR

Age-indexed eGFR and eGFR rate of decline are commonly used biomarkers for predicting rapid kidney function decline when aligned with cystic burden [5]. The European Renal Association-European Dialysis and Transplant Association (ERA-EDTA) defines rapid progression in ADPKD as an annual eGFR decline ≥3 mL/min over several years (due to eGFR fluctuations) [91,92]. eGFR decline often becomes apparent after significant kidney damage has occurred, at which point interventions to slow disease progression may be less effective [5]. Thus, eGFR alone may not reliably predict disease progression in the early stages. This is particularly challenging in younger patients where historical data are limited and variability in GFR estimation is greater in CKD G1-2 [5].

#### Total kidney volume (TKV)

Several methods are available for measuring TKV in ADPKD [93]. Planimetry, the gold standard, provide accurate measurements but require significant time and trained personnel [94–96]. The ellipsoid method offers a quicker alternative, though it may underestimate TKV in young patients [97]. Emerging automated methods, including deep learning-based segmentation, are demonstrating accuracy in TKV measurements [98]. The Consortium for Radiologic Imaging for the Study of Polycystic Kidney Disease (CRISP) study identified TKV as a key predictor of kidney function decline [79]. For example, a baseline TKV > 1500 mL was associated with a GFR decline of 4.33 ± 8.07 mL/min/year in 51 patients [79]. A later study confirmed that htTKV is a superior prognostic biomarker compared to age, serum creatinine, BUN, urinary albumin, and MCP-1 excretion [99]. These finding led to FDA approval TKV as a prognostic marker for kidney function decline in ADPKD, and its consideration as a reasonably likely surrogate endpoint in clinical trials [100,101].

#### Mayo imaging classification (MIC)

The MIC categorizes patients with ADPKD into five subclasses (1 A to 1E) based on htTKV and age. MIC predicts future eGFR and stratifies patients who are at risk of rapid progression [102]. Atypical cases of ADPKD are classified under class 2 (2 A with asymmetric disease and 2B with kidney atrophy) (supplemental Figure S4–S7). Patients with MIC 1 C to 1E are at high risk for reaching KF before age 65, making MIC essential for clinical decision making in those with rapidly progressing disease, especially regarding tolvaptan eligibility [5,103]. Obtaining serial htTKV measurements are recommended for patients who are borderline between Class 1B and 1 C [5]. Notably, most patients remain within their initial Mayo class over time [104]. Additionally, accurate measurements by planimetry are preferred when estimating MIC in patients younger than 25, as the small separation between classes means any underestimation by ellipsoid method could significantly alter risk stratification. Other limitations include the high cost for precise TKV measurements with CT and MRI, restriction to those aged between 15 and 80 years, limited applicability to ADPKD related to genes other than *PKD1* or *PKD2*, and lack of adequate representation of non-white populations in the initial study cohort which could limit generalizability [5].

#### The PROPKD score

The PROPKD score is a clinical tool that integrates clinical and genetic factors to predict the risk of KF before age 60 in ADPKD [105]. Points are assigned based on key variables: male sex (1 point), onset of hypertension before age 35 (2 points), a first urologic event (such as gross hematuria, cyst infection, or flank pain) before age 35 (2 points), and genetic variant type, with *PKD2* variants receiving 0 points, non-truncating *PKD1* variants 2 points, and truncating *PKD1* variants 4 points (supplemental Figure S5). The score ranges from 0 to 9. A score ≤3 indicate a low risk of progressing to KF before age 60, with a negative predictive value (NPV) of 81.4%. In contrast, scores >6 suggest a rapid progression toward KF before age 60, with a positive predictive value (PPV) of 90.9%. Scores in the intermediate range (4 to 6) indicate an uncertain prognosis [105]. The PROPKD score offers a strong predictive value for rapid progressors, particularly when MIC estimates are inconclusive or eGFR decline is unclear [7]. However, it is limited by the availability of genetic testing, which is not routinely performed in all ADPKD patients [5]. Another drawback is its intermediate-risk category (score 4–6), which lacks certainty, making clinical decision-making more challenging. Additionally, the PROPKD score is less applicable to younger patients (<35 years) unless they are hypertensive or have had early urologic events [7]. Both MIC and the PROPKD score are valuable tools for risk stratification in ADPKD, but they serve different roles and have distinct advantages and limitation [106]. A recent study explored the limitation both tools, particularly in intermediate-risk patients and their combined utility for better risk stratification in ADPKD. While combining these scores improved specificity, it had lower sensitivity and did not significantly enhance overall predictive power. However, reclassification using both scores identified a subgroup of intermediate-risk patients (38%) who may benefit from early tolvaptan treatment, yet only 60% were receiving therapy, indicating a potential gap in clinical management [106,107].

#### Advanced imaging biomarkers

Imaging texture analysis shows promise as a method for identifying rapid progression in kidney disorders. Kline et al. demonstrated that texture features, including entropy, gradient, contrast, dissimilarity, homogeneity, energy, correlation, and Angular Second Moment (ASM), extracted from T2-weighted MRIs significantly enhanced predictive models when combined with age and eGFR [108]. Cyst segmentations are also promising tools to assess ADPKD progression. Strong associations have been identified between kidney function decline and advanced imaging biomarkers, such as total cyst volume, renal parenchymal volume, and total cyst number [109]. These biomarkers have not yet been implemented in routine clinical practice.

## Clinical manifestations, complications and management of ADPKD

**Figure 1.**
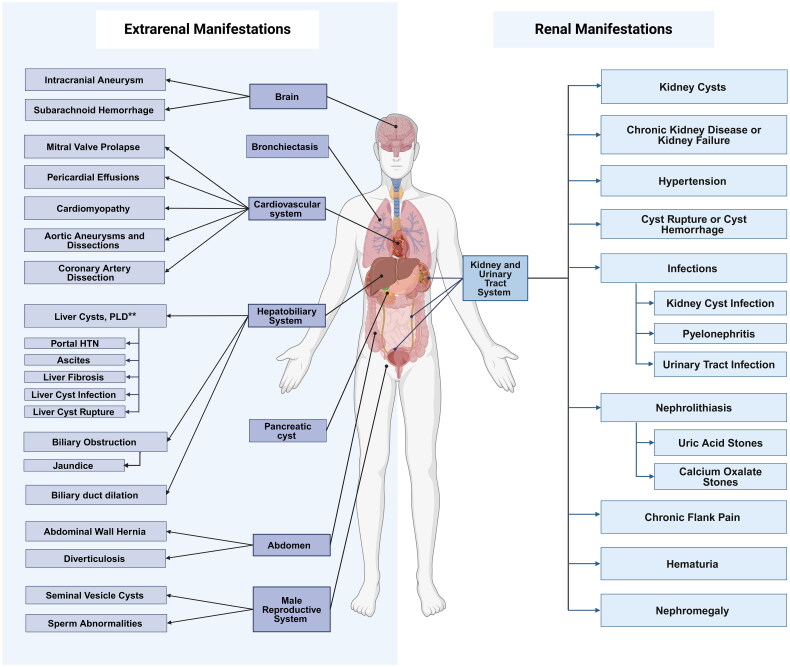
Distribution of the different renal and extrarenal manifestations in ADPKD: This figure illustrates the renal and extrarenal manifestations of ADPKD. Extrarenal manifestations include the Central nervous system (intracranial aneurysms, subarachnoid hemorrhages), the cardiovascular system (valve abnormalities, cardiomyopathy, aortic dissections), the hepatobiliary system (**) (over 80% of patients with ADPKD developing polycystic liver disease (PLD) and 17% having biliary duct dilation. Other complications such as portal hypertension, ascites, and jaundice are relatively rare and are due to the mechanical effect of liver cysts [110–112]). additional findings include pancreatic cysts, abdominal wall hernias, diverticulosis, and reproductive abnormalities (e.g., seminal vesicle cysts, sperm issues). Renal manifestations include kidney cysts, hypertension, nephrolithiasis, kidney infections, and cyst ruptures, with eventual progression to kidney failure. The disease often causes chronic flank pain, hematuria, and nephromegaly.

ADPKD presents with a wide spectrum of renal and extrarenal manifestations (Figure 1), with most patients being asymptomatic until the third decade of life (Table 2). Below is an overview of key manifestations, complications, and their management:

**Table 2.** Major characteristics of renal and extrarenal manifestations in ADPKD.

Renal manifestations				
	Prevalence	Typical presentation	Comorbidity/Complications	Special considerations/screening
CKD/KF	−98% of male and 77% of female patients reach kidney failure by age 80. -Two-third of patients with typical ADPKD are estimated to be rapid progressors	-Early stages: Decreased urinary concentration ability, low-grade proteinuria. -Late stages: High serum creatinine on routine blood test, Uremic signs or symptoms	- Kidney failure necessitating kidney transplant or dialysis. - Peritoneal dialysis is not contraindicated [113].	- Assess disease severity and risk of rapid progression by TKV, Mayo imaging class or annual GFR rate of decline. - Referral to nephrology clinic to consider disease-modifying treatments such as tolvaptan, or clinical trials. - Evaluate other kidney disease etiologies if there is an acute drop in GFR - Discuss the inherited nature of the disease to alert first-degree relatives to consider renal ultrasound for screening
Hypertension	- Up to 20% in children and adolescents − 50–70% before the age 30 despite preserved kidney function − 75–82% as CKD progresses	-During routine examination or vital signs check in urgent care -Headache	Left ventricular hypertrophy, stroke, vascular dissections, worsening kidney function, risk factor in development and rupture of intracranial aneurysm	- Recommend blood pressure monitoring in young individuals with family history of ADPKD [114]. - ACEI or ARB as first line therapy - Age 18–49, GFR >60 ml/min/1.73m^2^: BP goal ≤ 110/75 mm Hg - Age ≥ 50, any GFR: Systolic BP goal < 120 mm Hg
Cyst rupture or gross hematuria	up to 40%*	Acute flank or abdominal pain Less frequently gross hematuria	Risk of ureteral obstruction or acute kidney injury Worse kidney survival if early onset (<age 35), or if recurrent	- Typically, symptoms spontaneously resolve within a week. Oral or IV hydration with pain control might be needed. - In cases of severe and persistent hemorrhage, renal artery embolization, tranexamic acid, or rarely, nephrectomy might be needed [115,116].
Kidney cyst infection, pyelonephritis or UTIs	Up to 30%*	Abdominal pain, dysuria, fever, elevated C-reactive protein	Abscesses, emphysematous pyelonephritis, or severe recurrent infections necessitating a nephrectomy	- Urine culture and blood culture before antibiotic therapy if suspecting cyst infection. - CT of the abdomen, PET/CT scan, or indium WBC scans to identify the infected liver or kidney cyst. - Prolonged course of antibiotics with good tissue penetration when treating cyst infection - Consider cyst aspiration of the infected cyst if no clinical improvement with antibiotics
Chronic flank or abdominal pain	Up to 60%*	Abdominal or flank pain	Decreased quality of life, productivity, psychosocial distress	Chronic pain does not always correlate with TKV. Can be caused by renal capsule distention and aggravated by mechanical back pain
Nephrolithiasis	Up to 50%*	Kidney colic pain, abdominal discomfort, hematuria	Obstructive uropathy, stone infection	- Uric acid stones are more common in ADPKD (50%) compared to general population (<10%); remainder of stones are calcium oxalate. - CT scan is the preferred imaging modality given low sensitivity of MRI in detecting nephrolithiasis. Dual energy CT when available to differentiate calcium from uric acid stones. − 24-hour urine supersaturation to assess modifiable risk factors
Extrarenal manifestations in ADPKD				
	Prevalence	Typical presentation	Comorbidity/Complications	Special considerations/screening
Liver cysts, Polycystic liver disease (PLD) [117–119]	Common (>80% have liver cysts by age 30). Severe and symptomatic disease less common (< 5%)	Abdominal fullness, abdominal pain, severe heartburn, early satiety, dyspnea, weight loss/malnutrition	Pain, cyst infection, hemorrhage, compression of IVC, portal vein and hepatic veins	- Assessment of liver cystic disease presence and burden at initial and subsequent visits - Classify severity of liver cystic disease based on cystic burden and assess symptoms to guide treatment - Avoid or limit exogenous estrogen in females with moderate or severe disease (Schenldolffer/Mayo type C or D) - Growth in liver volume may decline after menopause
Liver cyst infection	Infrequent	Fever, abdominal pain, leukocytosis, high C-reactive protein	Liver abscess, sepsis if untreated	Given high risk of morbidity, antibiotics should be started empirically after obtaining blood cultures - PET/CT scan to localize infected cyst - Treatment options include prolonged antibiotics course and drainage of infected cysts
Intracranial aneurysms (IA)	9.2–18.5% with increased prevalence if positive family history of IA or SAH	-Asymptomatic when diagnosed during screening -Thunderclap (severe) headache due to IA rupture and subarachnoid hemorrhage (SAH)	High morbidity and mortality of >50% in case of ruptured aneurysm	-Screening is recommended in patients with high-risk profile (family history of IA, SAH or unexplained sudden death) or high-risk occupation (pilot, bus driver, etc.), or prior to major elective surgery such as kidney transplant or hepatic resection - Screening using brain magnetic resonance angiography without gadolinium (time-of-flight MRA), or CT head with IV contrast as an alternative. - If no IA detected, screening every 5 years in high-risk profiles or every 10 years for all others after discussing risks/benefits of screening
Mitral valves prolapse	3–26%	Mostly asymptomatic, may not have an audible murmur.	Mitral regurgitation, and its hemodynamic consequences. Arrythmias	Echocardiography if signs of cardiac dysfunction or heart murmur.
Pericardial effusions [120,121]	Up to 20%*, independent of kidney function	Usually, asymptomatic	Very rarely cardiac tamponade	-No role for screening, incidental diagnosis
Cardiomyopathy [122]	Up to 8%*			More commonly associated with *PKD2*.
Thoracic or aortic aneurysms and dissections, coronary artery dissection	Rare-1.5%	Acute chest or back pain, acute coronary syndrome presentation	Myocardial ischemia, Shock, death if untreated	- No role for additional screening outside of USPSTF guidelines [123] in patients without family history -In case of family history of aortic root, thoracic aortic aneurysms, or coronary artery dissection [124], first degree relatives should be screened
Abdominal wall hernia [125–127]	45% ADPKD with kidney failure	Bulge, swelling, discomfort, pain	Rarely bowel strangulation or incarceration	-Nonsurgical approach if asymptomatic -Surgical repair in people who elect for peritoneal dialysis or sometimes kidney transplantations

*Lifetime risk.

CKD: Chronic Kidney Disease, KF: Kidney failure, ADPKD: Autosomal Dominant Polycystic Kidney Disease, TKV: Total Kidney Volume, GFR: Glomerular Filtration Rate, BP: Blood Pressure, ACEI: Angiotensin-Converting Enzyme Inhibitor, ARB: Angiotensin II Receptor Blocker, UTI: Urinary Tract Infection, CT: Computed Tomography, PET: Positron Emission Tomography, PLD: Polycystic Liver Disease, MRA: Magnetic Resonance Angiography.

## A-kidney manifestations

### Cyst development, decreased eGFR, and kidney failure

Cystogenesis begins *in utero* but typically remains asymptomatic until early adulthood [42]. Kidney volume, particularly ht-TKV, is a strong predictor of disease progression; an ht-TKV >600 mL/m is associated with CKD stage 3 within eight years [128]. Reduced concentrating ability and elevated vasopressin levels contribute to cystogenesis and KF [129,130]. Once kidney function declines, the GFR decreases by 2–5 mL/min/year, depending on age and disease severity, with half of ADPKD patients reaching KF by age 60 [128].

Disease progression is strongly influenced by genotype. Patients with *PKD1*T (truncating) have the most severe phenotype, reaching KF at a median age of 55.6 years. *PKD1NT* (nontruncating) variants result in a milder course, with KF occurring at 67.9 years, while *PKD2* variants are associated with milder phenotype, developing KF around 79.7 years [128]. The minor and newer pathogenic variants associated with ADPKD (Table 1) have distinct phenotypes that overlap with ADPKD-*PKD1* and ADPKD-*PKD2* [7]. For instance, ADPKD-*IFT140* is typically associated with increased TKV due to a few large cysts but has a low risk of kidney failure [21]. ADPKD-*DNAJB11* involves only a few small cysts without TKV increase but carries a high risk of kidney failure later in life due to fibrosis [131]. Interestingly, bilateral cysts involving three or more cysts are commonly observed in collagenopathies, such as those associated with variants in *COL4A3* and *COL4A4*. These phenotypes may resemble features seen in rare ADPKD-associated genes like *IFT140*, *DNAJB11*, *GANAB*, *ALG5*, *ALG8*, and *ALG9*, suggesting overlapping genetic and phenotypic mechanisms [132].

Management of ADPKD should start with prevention and lifestyle modifications [7]. Hydration, with up to 3 liters of water daily, is recommended to reduce vasopressin activity and manage kidney stones risks. However, despite being shown to reduce urine osmolality, increased water intake has not been shown to significantly slow eGFR decline or slow the TKV growth over three years [133].

For patients progressing to KF, living donor kidney transplantation is preferred due to better long-term outcomes. Hemodialysis or peritoneal dialysis is recommended as a bridge to transplantation or when transplantation is not feasible [100,134]. Nephrectomy may be necessary if there is history of recurrent cyst infections or bleeding, severe chronic pain, or suspected malignancy or to make space for transplant in select cases [77,135].

**Figure 2.**
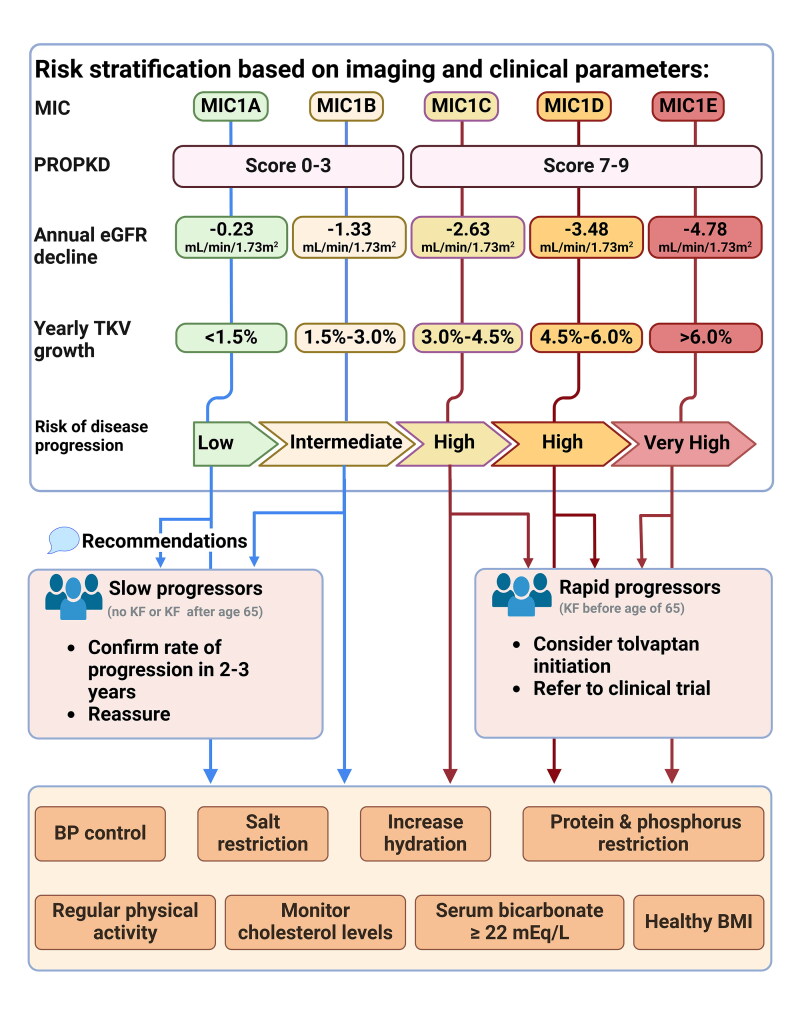
Management strategy of ADPKD patients based on risk stratification based on imaging and clinical parameters: This figure presents a risk strati­fication and management algorithm for patients with autosomal dominant polycystic kidney disease (ADPKD), helping clinicians distinguish slow progressors (MIC 1 A/1B, PROPKD 0–3) from rapid progressors (MIC 1 C/1D/1E, PROPKD 7–9). Slow progressors, unlikely to develop kidney failure (KF) before age 65, exhibit minimal decline in glomerular filtration rate (GFR) and total kidney volume (TKV) growth and are monitored every 2–3 years. Rapid progressors, at risk of KF before age 62, demonstrate faster GFR decline and TKV growth and are candidates for clinical trials or tolvaptan therapy. All ADPKD patients should adopt lifestyle changes, including blood pressure control, dietary modifications, and physical activity, to mitigate disease progression.

Tolvaptan, a selective V2R antagonist, has demonstrated efficacy in slowing disease progression by inhibiting cyst growth (Figure 2) [136–138]. In the TEMPO 3:4 trial, tolvaptan reduced kidney function decline by 30% reduction and decreased kidney growth rate by 49% decrease in kidney growth rate over three years in patients with TKV >750 mL and creatinine clearance (CrCl) > 60 mL/min/1.73m^2^ [139]. Similarly, the REPRISE trial showed that tolvaptan slowed the decline in eGFR by 35% over one year in patients with more advanced CKD (CrCl of 25–65 mL/min/1.73m^2^) [140]. An analysis by Torres et al. on CKD G4 patients in the open label trial (OLE) demonstrated that switching from placebo to tolvaptan significantly slowed eGFR decline, with benefits extending to those with lower eGFR (15–24 mL/min/1.73 m^2^) [141]. Therefore, tolvaptan remains effective even at eGFR levels as low as 15 mL/min and is recommended until KRT is required [5]. The most common side effects of tolvaptan include polyuria, excessive thirst, and nocturia. Liver function monitoring is essential due to the risk of reversible hepatoxicity [103]. The effect of tolvaptan is sustained and cumulative and thus it is recommended to start as early as able in adult patients with ADPKD at risk of rapid progression [5]. The drug is contraindicated in pregnancy, lactation, history of liver injury, hypovolemia, and urinary tract obstructions [141]. Supplemental Table S2 details clinical trials exploring various therapeutic agents in ADPKD.

### Hypertension

Hypertension, defined as a blood pressure reading above 130/80 mmHg, affects up to 70% of ADPKD patients by age 30, often preceding significant kidney function decline [4,142]. Blood pressure (BP) control is a key component of management, with targets tailored to patient age and kidney function. For most patients, maintaining BP <120/80 mm Hg is recommended. However, in patients aged 18 to 50 with an eGFR >60 mL/min/1.73m^2^, a stricter target of <110/75 mmHg is advised. The etiology of hypertension in ADPKD is multifactorial, involving activation of the renin-angiotensin-aldosterone system (RAAS), increased sympathetic activity, and vascular dysfunction mediated by endothelin and nitric oxide imbalances [143]. Early detection and management of hypertension are important, as cardiovascular disease remains the leading cause of death in ADPKD [1]. First-line therapy typically includes angiotensin-converting enzyme inhibitors (ACEIs) or angiotensin II receptor blockers (ARBs). Dual RAAS blockade is avoided due to the increased risk of hyperkalemia and the lack of additional benefit as shown in HALT-PKD trials [7,129, 142,144].

### Abdominal pain

Abdominal pain, originating from the kidneys or liver, is a common symptom in ADPKD, affecting about 50% of patients [145]. Acute pain may result from urinary tract infections, cyst infections, or kidney stones [146]. Non-PKD specific causes, such as peptic ulcer disease, pancreatitis, appendicitis, irritable bowel syndrome, and gynecological conditions, should be included in the differential diagnosis [147]. Chronic pain in ADPKD is primarily caused by cyst growth, which stretches the kidney capsule or enlarges the liver, compressing nearby structures. This pain can significantly impair quality of life [4,143,148]. Management often begins with noninvasive strategies, such as ice massages, heating pads, physical therapy, and techniques like the Alexander method [149]. Acetaminophen is typically the first-line analgesic, with gabapentin or other adjuvant medications as additional options. Nonsteroidal anti-inflammatory drugs (NSAIDs) may be used cautiously for short durations in patients with preserved kidney function. Opioids can be considered in severe cases [150,151]. For larger cysts causing significant pain, percutaneous aspiration and/or sclerotherapy may provide relief [152]. Invasive interventions, including celiac nerve blockade, radiofrequency ablation, spinal cord stimulation, and laparoscopic cyst fenestration, are reserved for refractory cases [145,150,151]. Nephrectomy or partial hepatectomy may be considered as a last resort for patients with debilitating pain unresponsive to other therapies [145,150,151].

### Cyst infections and urinary tract infection (UTI)

UTIs are common in ADPKD, affecting 30 to 50% of patients, with women being at higher risk, most often caused by gram-negative bacteria [1]. Clinical signs, including abdominal or flank pain, fever, and elevated inflammatory markers, should prompt consideration of a cyst infection [153]. Diagnostic evaluation includes urinalysis and urine culture with sensitivity testing. Blood cultures are warranted in cases of systemic symptoms. Imaging, such as CT or MRI, is employed to localize the infection, although white blood cell indium scanning or 18-fludeoxyglucose positron emission tomography (18 FDG-PET) provides greater sensitivity when the source is unclear [154,155].

Treatment begins with empiric broad-spectrum antibiotics, which are tailored based on culture results. Antibiotics like trimethoprim-sulfamethoxazole or fluoroquinolones are preferred due to their superior cyst penetration, whereas beta-lactams might be less effective for cyst infections [156]. The duration of therapy depends on the infection type. Cyst infections require extended courses of 4–6 weeks. Persistent symptoms despite antibiotic therapy may require CT-guided aspiration for diagnosis and drainage [1,153, 156,157].

### Cyst hemorrhage and hematuria

Cyst hemorrhage is a frequent occurrence in APDKD, particularly among patients on dialysis, and the likelihood often correlates with TKV [1,158]. Hemorrhagic cysts are identified on imaging as high-density lesions. Cysts may rupture, resulting in bleeding confined within the cyst, communication with the collecting system, or in rare cases extension into the subscapular or retroperitoneal space [158]. Hematuria can range from microscopic to gross, with the latter being more common in patients with larger kidneys [3,116]. Gross hematuria presenting at a younger age is associated with a worse prognosis [76,148,159].

Management of cyst hemorrhage is predominantly conservative, focusing on rest and hydration. In severe cases, suspending ACEIs or ARBs may help mitigate the risk of acute kidney injury [160,161]. Blood transfusions, if required, should be administered cautiously to avoid sensitization, which could complicate future kidney transplantation. Interventional radiology-guided embolization is indicated for refractory bleeding or hemodynamic instability. Tranexamic acid, an antifibrinolytic medication, may be used on select cases to control persistent bleedings. Unilateral nephrectomy is a last-resort intervention, rarely required to treat severe refractory cyst bleeding [115,116].

### Nephrolithiasis

The prevalence of kidney stones in people with ADPKD ranges from 3% to 59% [162]. Uric acid stones are the most common type, followed by calcium oxalate stones [159,163]. Both anatomic distortions of the kidneys and metabolic factors, such as hypocitraturia and lower urine pH, contribute to the increased risk of stone formation [164,165]. Dual-energy computed tomography (DECT) is the most accurate diagnostic tool for differentiating uric acid stones from other renal stones, achieving 100% sensitivity and specificity [166].

Management begins with ensuring adequate hydration to dilute urine, along with appropriate pain control [167,168]. For obstructive or complex stones, interventions such as extracorporeal shock wave lithotripsy (ESWL), percutaneous nephrolithotomy, and flexible ureteroscopy are often required [167–169]. However, the presence of large cysts in ADPKD complicate ESWL or percutaneous nephrostomy [170], making flexible ureteroscopy a preferred option due to its safety and expedited recovery [167–169]. Preventive strategies are important to reducing recurrence risk and include addressing metabolic abnormalities identified through 24-h urinary testing or stone analysis. Key recommendations include maintaining a high fluid intake (>3 liters/day to achieve a urine output >2.5 liters), consuming a diet restricted in sodium and animal proteins, and correcting specific metabolic derangements. Potassium citrate is effective for hypocitraturia, while allopurinol or febuxostat may be considered for managing hyperuricemia associated with uric acid stones, although their use in this context is not universally accepted [171].

### Renal cell carcinoma (RCC)

RCC is rare in ADPKD, occurring in <1% of cases, a prevalence comparable to other kidney diseases. However, RCC in ADPKD often presents at earlier age and is frequently bilateral, multicentric, and of the sarcomatoid type. Systemic symptoms, including fever, anorexia, weight loss, and fatigue, should raise suspicion. Imaging findings indicative of RCC include the rapid growth of complex cysts, speckled calcifications, local lymphadenopathy, and thrombi, which are best visualized on CT scans. While ultrasound (US) may detect complex cysts, CT imaging remains the diagnostic modality of choice for evaluating suspicious lesions. Despite its earlier and more aggressive presentation in ADPKD, routine RCC screening is not recommended due to the low overall incidence [172].

## B-extrarenal manifestations

### Extrarenal cysts

Liver cysts are the most common external manifestation of ADPKD, with prevalence increasing with age, reaching about 80–90% after age 35 [1,4]. Risk factors for a higher liver cystic burden include estrogen-containing birth control, hormone replacement therapy, and greater number of pregnancies [173]. Polycystic liver disease (PLD) may impact quality of life, causing abdominal pain, bloating, early satiety, and dyspnea due to diaphragm compression [1,4]. Complications such as cyst hemorrhage and infection can be detected by CT or MRI [174]. Management is tailored to severity and includes pain relief, cyst aspiration with sclerotherapy, cyst fenestration, partial hepatectomy, or liver transplantation [76,174]. Pancreatic cysts are less common (19% of patients) and are rarely symptomatic [175]. Seminal vesicle cysts occur in about 40% of male patients with ADPKD [176].

### Cardiac manifestations

Cardiovascular disease is the leading cause of death among patients with ADPKD [144]. Left ventricular hypertrophy (LVH) is common, increasing the risk of arrhythmias and heart failure [144]. Historical studies reported LVH prevalence of 65% in ADPKD, compared to 55% in controls [177], but more recent studies, including HALT-PKD trial, show significantly lower rates (3.9%) following the widespread RAAS-blockers use [178]. Additionally, at kidney transplantation, patients with ADPKD exhibit lower LVH and LV mass compared to non-ADPKD patients [179]. Management of LVH includes strict blood pressure control and RAAS blockade [180], with monitoring *via* echocardiograms and strain imaging to assess ejection fraction [181]. Mitral valve prolapse affects up to 21% of ADPKD patients [182], and echocardiography is recommended for those with heart murmurs [183]. *PKD1* variants may increase the risk for congenital heart defects [184].

### Pulmonary manifestations

Patients with ADPKD have a threefold increased risk of bronchiectasis compared to other CKD patients, potentially due to the dysfunctional PC1 and PC2 in the motile airway epithelial cilia or bronchial smooth muscles cells [185,186].

### Gastrointestinal manifestations

Diverticular disease, involving intestinal smooth muscle dysfunction, may arise secondary to PC1 and PC2 defects [187]. Patients with ADPKD who reached KF are at increased risk of diverticulitis or colon perforation [187,188].

### Intracranial aneurysm (IAs)

IAs occur in 8–12% of patients with ADPKD, compared to 3.2% in the general population [189,190], and prevalence rises to 22% in those with a family history of IAs or subarachnoid hemorrhage (SAH) [189,191]. Most IAs in ADPKD are small, saccular, and located in the anterior brain circulation [190]. Key nonmodifiable risk factors for IAs include a family or personal history of IAs or SAH, female sex, and older age [192,193]. Modifiable risk factors include smoking, chronic alcohol use, hyperlipidemia, and uncontrolled hypertension [194]. Screening for IAs is best performed by using time-of-flight non-contrast magnetic resonance angiography (MRA). While no consensus exists on screening strategy, most experts favor targeted screening for individuals with risk factors, particularly family history of IA or SAH [195,196]. Ruptured IAs lead to SAH, a life-threatening condition often preceded by sentinel or thunderclap headaches, with a case fatality rate of approximately 35% [197,198].

Management of unruptured IAs depends on age, aneurysm size, and symptoms. Larger or symptomatic IAs in younger patients often require intervention, while smaller aneurysms (<7 mm) in older patients are generally managed with monitoring *via* serial MRA [199–201]. Initial care involves a multidisciplinary team, including nephrologists, vascular neurologists, and neurosurgeons [202]. For SAH, key treatments include continuous monitoring, blood pressure control, pain relief, thromboprophylaxis and antiepileptic drugs [203]. The choice between endovascular and microsurgical repair is patient-specific [204].

## C-ADPKD in children

### Diagnosis of ADPKD in children

Although ADPKD typically manifests in adulthood, some individuals develop early-onset disease [39]. The ADPKD spectrum includes typical adult-onset manifestations, early-onset ADPKD (ADPKD_EO_, before age 15), and very early onset ADPKD (ADPKD_VEO_, diagnosed *in utero* or before 18 months) [205]. Diagnostic criteria for ADPKD_VEO_ *in utero* include oligohydramnios and hyperechoic, enlarged kidneys (> 2 standard deviation). For infants up to 18 months, diagnosis requires enlarged, palpable kidneys plus ≥1criterion: blood pressure above the 95^th^ percentile (or antihypertensive use), GFR <90 mL/min/1.73m^2^, or persistent proteinuria [34]. ADPKD_EO_ diagnosis (18 months to 15 years) requires at least one of the following: enlarged, palpable kidneys, elevated blood pressure, decreased GFR, or persistent proteinuria [34]. US is the preferred screening method for at-risk children, but its use should consider the medical, legal, and psychological implications of an ADPKD diagnosis. Detection one or more kidney cysts in a child with a positive family history is highly suggestive of ADPKD. When results are equivocal follow-up studies are required [206]. MRI offers higher sensitivity but is often avoided in younger children due to sedation requirements [42]. Genetic testing is recommended for atypical or early-onset presentations and cases without family history of cystic kidneys [42]. ADPKD_VEO_ is linked to reduced gene dosage, often from biallelic *PKD1* variants, particularly hypomorphic types [207]. The decision to screen children under age 18 remains controversial. Early diagnosis may aid blood pressure control, adopt additional measures for healthy lifestyle, avoidance of nephrotoxins, and kidney function monitoring; but concerns about the psychologic and social burdens persist [206,208].

### Manifestations of ADPKD in children

Nonspecific symptoms in children include abdominal, flank, or back pain, cyst infections or bleeding [209]. Gross hematuria occurs in 10–14% of children before age 16, while symptoms like polyuria, urinary frequency, and enuresis (due to reduced urine concentrating ability) are seen in around 58% [209–211]. Hypertension and mild proteinuria are common with 20–40% of children affected [210,212,213]. Hypertension accelerates kidney function decline and kidney growth [214,215]. Children with ADPKD_VEO_ face a higher risk for kidney function loss at a younger age [216]. While most children maintain adequate kidney function into their 30s [217], severe neonatal presentations can resemble ARPKD [218]. *NEK8* variants, particularly biallelic pathogenic variants, are linked to severe syndromic ciliopathies, whereas monoallelic variants in the kinase domain primarily affect the kidneys and resemble ADPKD_VEO_ [25,219].

### Management of ADPKD in children

Asymptomatic patients typically do not require treatment until adulthood, but early intervention is essential for those with hypertension or other symptoms [42]. Tolvaptan use in children is under investigation. A recent phase 3 trial suggest it slows kidney volume growth and eGFR decline, though statistical significance was not achieved due to small sample sizes and short study durations [220]. Ambulatory blood pressure monitoring (ABPM) is preferred for diagnosing hypertension in children aged ≥5, as it detects isolated nocturnal hypertension [221]. RAAS inhibitors are recommended to control blood pressure and slow kidney function decline [222,223]. Maintaining a healthy weight and reducing salt intake are additional strategies for optimal disease management [224]. Pravastatin was evaluated in a trial involving children and young adults (ages 8–22) with ADPKD [225]. Over three years, it significantly reduced HtTKV growth, although it had no impact on left ventricular mass index, urine microalbumin excretion, or kidney function [225].

### Leuven imaging classification

*A* Belgian study proposed using htTKV measured *via* 3D ultrasound for pediatric ADPKD patients younger than 19 years old [226]. The MIC model often underestimated disease severity, especially in children under age 10 [102] Adjustments led to a new predictive model based on htTKV, which provided more accurate risk stratification of pediatric patients. Validation with Mayo Clinic and CRISP data confirmed the new model’s superiority in risk assessment compared to the MIC model [226].

## D-Family planning, pregnancy, and lactation in ADPKD

Women with ADPKD should be informed of potential worsening of their liver cystic burden due to estrogen exposure, especially with moderate to severe PLD. Multiple pregnancies (>3) are associated with faster GFR decline in ADPKD patients [1,227]. Estrogen-containing contraceptives are associated with liver cyst growth and the progression of PLD, particularly with prolonged use [228]. Combined hormonal contraceptives (estrogen and progestin), especially those containing low levels of estrogen, are generally preferred and can be used in ADPKD patients with or without mild PLD [7,229]. Preimplantation genetic testing for monogenic disorders (PGT-M) allows affected couples to have children without passing on the disease [88]. RAAS inhibitors should be avoided during pregnancy. Instead, labetalol, nifedipine, or alpha methyl dopa are preferred. Women with normal blood pressure and kidney function generally have favorable pregnancies but face increased risks of pregnancy-induced hypertension and preeclampsia [230,231]. Those with reduced kidney function require close monitoring by nephrologists and high-risk pregnancy specialists due to risks of fetal loss, kidney decline, and blood pressure challenges [227]. Screening for IAs during pregnancy in ADPKD is not routinely indicated but may be considered before pregnancy for those with a positive family history, extracranial vascular conditions, or *de novo* ADPKD [227,232]. Special considerations are also needed in lactating females. Most antihypertensive agents are excreted in very low amounts in the breast milk and are generally safe for use. Suitable medications include alpha methyl dopa, nifedipine, verapamil, metoprolol, propranolol, hydrochlorothiazide (≤50 mg daily), enalapril, and benazepril. However, data on ACEIs and ARBs are limited, and labetalol use requires caution in preterm babies [233].

## Conclusion

ADPKD is a systemic multiorgan disorder that profoundly impacts kidney and extrarenal function, significantly affecting quality of life. Advances in diagnostic tools, such as genetic testing and imaging, have enhanced early detection and improved accuracy of risk prediction [90]. While tolvaptan slows kidney disease progression in adults with ADPKD at risk of rapid progression, there is an urgent need for therapies to halt or reverse cyst growth. Future research should prioritize individualized treatments tailored to genetic and molecular characteristics.

## Supplementary Material

Supplemental Material

SupFig1E.jpg

SupFig7C.jpg

SupFig6.jpg

SupFig3B.jpg

SupFig5B.jpg

SupFig1H.jpg

SupFig2B.jpg

SupFig1D.jpg

SupFig1K.jpg

SupFig1M.jpg

SupFig1G.jpg

SupFig7A.jpg

SupFig1A.jpg

SupFig5A.jpg

SupFig7B .jpg

SupFig1P.jpg

SupFig 4.jpg

SupFig 1i.jpeg

SupFig1O.jpg

SupFig1J.jpg

SupFig3A.jpg

SupFig1N.jpg

SupFig1B.jpg

SupFig1F.jpg

SupFig2A.jpg

SupFig1C.jpg

SupFig1L.jpg
