# Supplementary material for: Autosomal dominant polycystic kidney disease: an overview of recent genetic and clinical advances
Source: Ren Fail. 2025 Apr 23;47(1):2492374. doi: 10.1080/0886022X.2025.2492374 (PMC12020221; doi:10.1080/0886022X.2025.2492374)
Supplement: Supplemental Material [file IRNF_A_2492374_SM2537.docx]

**References:**1- Kline TL, Edwards ME, Fetzer J, Gregory AV, Anaam D, Metzger AJ, et al. Automatic semantic segmentation of kidney cysts in MR images of patients affected by autosomal-dominant polycystic kidney disease. Abdom Radiol (NY). 2021;46(3):1053-61

2- Kline TL, Korfiatis P, Edwards ME, Bae KT, Yu A, Chapman AB, et al. Image texture features predict renal function decline in patients with autosomal dominant polycystic kidney disease. Kidney Int. 2017;92(5):1206-16

Walz G, Budde K, Mannaa M, Nurnberger J, Wanner C, Sommerer C, et al. Everolimus in patients with autosomal dominant polycystic kidney disease. N Engl J Med. 2010;363(9):830-40.

3- Serra AL, Poster D, Kistler AD, Krauer F, Raina S, Young J, et al. Sirolimus and kidney growth in autosomal dominant polycystic kidney disease. N Engl J Med. 2010;363(9):820-9.

4- de Lemos Barbosa CM, Souza-Menezes J, Amaral AG, Onuchic LF, Cebotaru L, Guggino WB, et al. Regulation of CFTR Expression and Arginine Vasopressin Activity Are Dependent on Polycystin-1 in Kidney-Derived Cells. Cell Physiol Biochem. 2016;38(1):28-39.

5- Yanda MK, Cebotaru L. VX-809 mitigates disease in a mouse model of autosomal dominant polycystic kidney disease bearing the R3277C human mutation. FASEB J. 2021;35(11):e21987.

6- Takiar V, Nishio S, Seo-Mayer P, King JD, Jr., Li H, Zhang L, et al. Activating AMP-activated protein kinase (AMPK) slows renal cystogenesis. Proc Natl Acad Sci U S A. 2011;108(6):2462-7.

7- Liang D, Song Z, Liang W, Li Y, Liu S. Metformin inhibits TGF-beta 1-induced MCP-1 expression through BAMBI-mediated suppression of MEK/ERK1/2 signalling. Nephrology (Carlton). 2019;24(4):481-8.

8- Yheskel M, Patel V. Therapeutic microRNAs in polycystic kidney disease. Curr Opin Nephrol Hypertens. 2017;26(4):282-9.

9- Sweeney WE, Frost P, Avner ED. Tesevatinib ameliorates progression of polycystic kidney disease in rodent models of autosomal recessive polycystic kidney disease. World J Nephrol. 2017;6(4):188-200.

10- Parker MI, Nikonova AS, Sun D, Golemis EA. Proliferative signaling by ERBB proteins and RAF/MEK/ERK effectors in polycystic kidney disease. Cell Signal. 2020;67:109497.

11- Messchendorp AL, Casteleijn NF, Meijer E, Gansevoort RT. Somatostatin in renal physiology and autosomal dominant polycystic kidney disease. Nephrol Dial Transplant. 2020;35(8):1306-16.

12- Perico N, Ruggenenti P, Perna A, Caroli A, Trillini M, Sironi S, et al. Octreotide-LAR in later-stage autosomal dominant polycystic kidney disease (ALADIN 2): A randomized, double-blind, placebo-controlled, multicenter trial. PLoS Med. 2019;16(4):e1002777.

13- Meijer E, Drenth JP, d'Agnolo H, Casteleijn NF, de Fijter JW, Gevers TJ, et al. Rationale and design of the DIPAK 1 study: a randomized controlled clinical trial assessing the efficacy of lanreotide to Halt disease progression in autosomal dominant polycystic kidney disease. Am J Kidney Dis. 2014;63(3):446-55.

14- Peterschmitt MJ, Crawford NPS, Gaemers SJM, Ji AJ, Sharma J, Pham TT. Pharmacokinetics, Pharmacodynamics, Safety, and Tolerability of Oral Venglustat in Healthy Volunteers. Clin Pharmacol Drug Dev. 2021;10(1):86-98.

15- Qiu Z, He J, Shao G, Hu J, Li X, Zhou H, et al. Obacunone Retards Renal Cyst Development in Autosomal Dominant Polycystic Kidney Disease by Activating NRF2. Antioxidants (Basel). 2021;11(1).

16- Klawitter J, Zafar I, Klawitter J, Pennington AT, Klepacki J, Gitomer BY, et al. Effects of lovastatin treatment on the metabolic distributions in the Han:SPRD rat model of polycystic kidney disease. BMC Nephrol. 2013;14:165.

17- Gile RD, Cowley BD, Jr., Gattone VH, 2nd, O'Donnell MP, Swan SK, Grantham JJ. Effect of lovastatin on the development of polycystic kidney disease in the Han:SPRD rat. Am J Kidney Dis. 1995;26(3):501-7.

18- Bais T, Gansevoort RT, Meijer E. Drugs in Clinical Development to Treat Autosomal Dominant Polycystic Kidney Disease. Drugs. 2022;82(10):1095-115.

19- Klawitter J, McFann K, Pennington AT, Wang W, Klawitter J, Christians U, et al. Pravastatin Therapy and Biomarker Changes in Children and Young Adults with Autosomal Dominant Polycystic Kidney Disease. Clin J Am Soc Nephrol. 2015;10(9):1534-41.

20- Saini AK, Saini R, Singh S. Autosomal dominant polycystic kidney disease and pioglitazone for its therapy: a comprehensive review with an emphasis on the molecular pathogenesis and pharmacological aspects. Mol Med. 2020;26(1):128.

21- Arkhipov SN, Potter DL, Sultanova RF, Ilatovskaya DV, Harris PC, Pavlov TS. Probenecid slows disease progression in a murine model of autosomal dominant polycystic kidney disease. Physiol Rep. 2023;11(7):e15652.

**Supplemental material:**

**Supplemental Tables:**

**Supplemental Table 1: Imaging findings on US, CT, or MRI to rule in or rule out ADPKD in the presence or absence of family history.**
Adapted from Chapman et al (Kidney Int. 2015;88:17-27)

**Supplemental Table 2: Overview of the mechanism of action and the preclinical findings and clinical findings of emerging treatments for autosomal dominant polycystic kidney disease (ADPKD)**

**Supplemental Figures:**

**Supplemental Fig. 1. Imaging representation (US, CT, or MRI) of ADPKD pathogenic variants**

Legend: Each image includes patient details such as age, sex, genetic variant, estimated glomerular filtration rate (GFR, mL/min/1.73m2), height-adjusted total kidney volume (HtTKV, mL/m), and Mayo imaging classification (MIC). The first row (A-D) shows patients with definite ADPKD, characterized by different levels of kidney enlargement and cyst development through MRI or CT scans. The second row (E-H) presents cases ranging from likely ADPKD (E), possible ADPKD (F), and definite ADPKD (G-H) with MRI (E-F-H) or Ultrasound (G). The third row (I-L) depicts less common ADPKD variants (e.g., IFT140, GANAB, DNAJB11, ALG8), characterized by atypical cyst patterns. The fourth row (M-P) shows patients with other cystic kidney diseases, including autosomal dominant tubulointerstitial kidney disease (ADTKD), ARPKD, and lithium-induced nephropathy. Image D illustrates the automated deep learning planimetry model used to measure kidney (red = right kidney, green = left kidney) and liver (red) volumes. This model calculates total kidney volume and total liver volume. Image H shows the automated cyst segmentation model, which labels each cyst with a different color. MR images and predicted kidney segmentations were input into the cyst segmentation model to produce individually labeled cysts. This model calculates total cyst number, total cyst volume, cyst parenchymal surface area, and other advanced imaging biomarkers using Python and the PyRadiomics library (1 ,2).

**Supplemental Fig. 2. Three-dimensional structure of polycystins proteins made by 1 PC1 and 3 PC2 proteins**

Legend: Schematic 2D representation (A) and 3D structure (B) of the polycystin complex composed of one polycystin-1 (PC1) and three polycystin-2 (PC2) subunits. (A) The two-dimensional PC1 and PC2 in the plasma membrane. PC1, encoded by the PKD1 gene, has 11 transmembrane domains and several functional domains, including the N-terminal domain (NTD), voltage sensor-like domain (VSD), and polycystin domain (PCD). PC2, encoded by the PKD2 gene, is represented with six transmembrane domains. (B) The three-dimensional structure of the PC1 complex in a stoichiometry of 1 PC1 to 3 PC2 subunits. The extracellular and intracellular regions of the polycystin complex are highlighted, demonstrating the arrangement of the voltage-sensing domains. This interaction between PC1 and PC2 is essential for the regulation of calcium signaling, prevention of cystogenesis, and stabilization of the proteins in cellular membranes. PLAT: Polycysting-1, Lipoxygenase, and α-Toxin Domain

Adapted from Su, Qiang, et al. "Structure of the human PKD1-PKD2 complex." Science 361.6406 (2018): eaat9819.

**Supplemental Fig. 3. Figure describing the maturation process of polycystins within the tubular epithelium along with possible pathways involved in ADPKD pathogenesis**

Legend:

(A): Maturation process of polycystin-1 (PC1) and polycystin-2 (PC2) through the endoplasmic reticulum and Golgi apparatus. N-linked glycosylation and protein folding are essential steps facilitated by genes such as GANAB, DNAJB11, ALG8, and PMM2. Proper endoplasmic reticulum translocation, mediated by SEC63, SEC51A1, and SEC61B, ensures that PC1 and PC2 reach the primary cilia, where they form the mature polycystin complex.

(B): The complex molecular cascade driving the progression of ADPKD. The disease is initiated by mutations in polycystin proteins, which disrupt normal calcium (Ca²⁺) and cyclic AMP (cAMP) signaling. Elevated levels of cAMP activate protein kinase A (PKA) and in turn, triggers a series of downstream effects, including the activation of the cystic fibrosis transmembrane conductance regulator (CFTR), which promotes chloride (Cl⁻) secretion, leading to fluid accumulation and cyst expansion. Moreover, PKA activation affects intracellular calcium balance by interacting with calcium channels, such as the IP3 receptor (IP3R) and Ryanodine receptor (RyR), leading to calcium mobilization. Overexpression of growth factors such as EGF, IGF1, and VEGF, further contributes to abnormal cell proliferation and cyst formation. Key pathways involved include the mTOR pathway, which is upregulated due to altered signaling. The PI3K/AKT and RAF/MEK/ERK pathways are also overactivated, promoting cell growth and proliferation. Additionally, transcription factors such as HIF, STAT3, and Nf-kB are dysregulated, further accelerating disease progression. Abnormal expressed microRNA (miRNA) downregulates key genes involved in proliferation, leading to decreased polycystin levels, cyst formation and disease progression. Therapeutic targets are focused on modulating cAMP signaling, calcium regulation, and inhibiting pathways such as mTOR and PI3K.

AC6: Adenylate Cyclase 6, AMPK: AMP-Activated Kinase, ATP: Adenosine Triphosphate; AVP: Vasopressin, CaMKK: Calcium/Calmodulin-dependent protein kinase, CDK: Cyclin-dependent kinase, EGF: Epidermal Growth Factor, ERK: Extracellular signal-regulated kinase, Gi: inhibitory G protein, Gq: G protein subunit, Gs: Stimulatory G protein, GSK3β: Glycogen synthase kinase 3β, HIF: Hypoxia-inducible factor, IGF1: Insulin Growth Factor 1, IP3R: Inositol 1,4,5-triphosphate receptor, LKB1: Liver kinase B1, MEK: Mitogen-activated protein kinase, mTOR: Mammalian Target of Rapamycin, PC1: Polycystin-1, PC2: Polycystin-2, P2R: Purinergic 2 receptor, PLC: Phospholipase C, Rheb: Ras Homolog enriched in brain, RyR: Ryanodine receptor, Sirt1: Sirtuin 1, SOC: Store-operated channel, STAT3: Signal transducer and activator of transcription 3, STIM1: Stromal interaction molecule 1, SSTR: Somatostatin receptor, TKIs: Tyrosine kinase inhibitors, TSC: Tuberous sclerosis proteins (TSC1/2), V2R: Vasopressin V2 receptor, CFTR: cystic fibrosis transmembrane conductance regulator, DVL: dishevelled segment polarity protein, AKT: Ak strain transforming, RSC: chromatin structure remodeling, ANO1: Anoctamin-1, PDE1: phosphodiesterase 1, AMP: adenosine monophosphate.

**Supplemental Fig. 4. Progression of kidney disease and symptoms development in ADPKD with Age**

This figure illustrates the natural progression of Autosomal Dominant Polycystic Kidney Disease (ADPKD) from normal kidney function to kidney failure, correlating disease severity with age, imaging classifications, and clinical manifestations. The graph shows the decline in estimated glomerular filtration rate (eGFR) across Mayo Imaging Classification (MIC) categories (1A to 1E), with more severe classifications (1C-1E) exhibiting steeper eGFR declines as total kidney volume (TKV) increases. The upper section depicts stages of disease progression (normal, hyperinflation, impairment, failure) in relation to age (15 to 60 years) and eGFR decline. The lower section highlights the onset and duration of chronic symptoms (e.g., urinary concentrating defects, hypertension, proteinuria, pain) and acute signs (e.g., hematuria, urinary tract infections, cyst ruptures, kidney stones) as the disease progresses. Adapted from Perumareddi, P. and D.P. Trelka, Autosomal Dominant Polycystic Kidney Disease. Prim Care, 2020. 47(4): p. 673-689.

**Supplemental Fig. 5. Predicting Disease Progression in ADPKD Using the Mayo imaging classification (MIC)**

A: The decline in estimated glomerular filtration rate (eGFR) over time (age in years) is shown for each MIC subclass. MIC1A to MIC1E represent progressive risk levels, with MIC1A indicating the slowest decline and MIC1E the fastest, correlating with disease severity.

B: Median age of kidney failure (KF) onset for each MIC subclass. MIC1A has the slowest progression to KF while MIC1E represents the highest risk group with the youngest median age of KF onset at 45.1 years.

Adapted from: Lavu, Sravanthi, et al. "The value of genotypic and imaging information to predict functional and structural outcomes in ADPKD." JCI insight 5.15 (2020).

**Supplemental Fig. 6. Progression of HtTKV with age in patients with different MIC**

The graph illustrates the relationship between Ht-TKV (mL/m) and age (years), with the five MIC subclasses (MIC1A to MIC1E) curves.

Adjacent images shows representative cases for each subclass, providing clinical details including age, GFR, Ht-TKV, and MRI findings. MIC1A represents the lowest risk, while MIC1E corresponds to the highest risk of disease progression.

**Supplemental Fig. 7. Predicting Disease Progression in ADPKD Using the PROPKD Score and Mayo Imaging Classification (MIC)**

Legend:

(A):. The graph shows the predicted increase in height-adjusted total kidney volume (Ht-TKV) over time, classified into MIC subclasses (MIC1A to MIC1E) in patients with ADPKD. The more severe the subclass (from MIC1A to MIC1E), the faster the increase in Ht-TKV, indicating more rapid disease progression.

(B): The PROPKD score system, which quantifies the risk of disease progression in ADPKD based on clinical and genetic factors. The scoring system considers sex, hypertension (HTN) and urologic complications before age 35, and the type of pathogenic variant in the PKD1 gene (either non-truncating [PKD1-NT] or truncating [PKD1-T]). Higher scores indicate a more severe disease progression.

(C): Age-related decline in estimated glomerular filtration rate (eGFR) across different MIC-1 classes. The curves illustrate the progression of kidney function [eGFR (mL/min/1.73 m²)] decline, at different rates, associated with different MIC-1 groups.

Adapted from: https://adpkdsim.org/expert/prognostic-tools/propkd-score

Cornec-Le Gall, Emilie, et al. "The PROPKD score: a new algorithm to predict renal survival in autosomal dominant polycystic kidney disease." Journal of the American Society of Nephrology 27.3 (2016): 942-951.

**Supplemental Table 1**: **Imaging findings on US, CT, or MRI to rule in or rule out ADPKD- *PKD1* or ADPKD-*PKD2* in the presence or absence of family history.**

Adapted from Chapman et al (Kidney Int. 2015;88:17-27)

| **Ultrasound-based** | | | | | | | | | | | | | | | | | | | | | | | | | |
| --- | --- | --- | --- | --- | --- | --- | --- | --- | --- | --- | --- | --- | --- | --- | --- | --- | --- | --- | --- | --- | --- | --- | --- | --- | --- |
| **ADPKD Diagnosis** | | | | | | | | | | | | | | | | | | | | | | | | | |
|  | | | ***PKD1* Pathogenic Variant** | | | | | | | | **PKD2 Pathogenic Variants** | | | | | | | **Unknown Familial Genotype** | | | | | | | |
| **Age** | **Criteria** | | | **Sensitivity** | | | | | | **PPV** | **Sensitivity** | | | **PPV** | | | **Sensitivity** | | | | | | | | **PPV** |
| 15-29 | ≥3 cysts (Total) | | | 94.3 | | | | | | 100 | 70 | | | 100 | | | 82 | | | | | | 100 | | |
| 30-39 | ≥3 cysts (Total) | | | 96.6 | | | | | | 100 | 95 | | | 100 | | | 96 | | | | | | 100 | | |
| 40-59 | ≥2 cysts/ kidney | | | 92.6 | | | | | | 100 | 89 | | | 100 | | | 90 | | | | | | 100 | | |
| >60 | ≥4 cysts/ kidney | | | 100 | | | | | | 100 | 100 | | | 100 | | | ND | | | | | | ND | | |
| **ADPKD Exclusion** | | | | | | | | | | | | | | | | | | | | | | | | | |
|  | |  | | | | | ***PKD1* Pathogenic Variant** | | | | | **PKD2 Pathogenic Variants** | | | | | | | **Unknown Familial Genotype** | | | | | | |
| **Age** | **Criteria** | | | | | | **Specificity** | | **NPV** | |  | | **Specificity** | | | **NPV** | | | | **Specificity** | |  | | **NPV** | |
| 15-29 | No Cysts | | | | | | 97.6 | 99.1 | | |  | | 96.6 | | 83.5 | | | | | 97.1 | | | | 90.8 | |
| 30-39 | No Cysts | | | | | | 96 | 100 | | |  | | 93.8 | | 96.8 | | | | | 94.8 | | | | 98.3 | |
| 40-59 | No Cysts | | | | | | 93.9 | 100 | | |  | | 93.7 | | 100 | | | | | 93.9 | | | | 100 | |
| **MRI or CT-based** | | | | | | | | | | | | | | | | | | | | | | | | | |
| **ADPKD Diagnosis** | | | | | | | | | | | | | | | | | | | | | | | | | |
| **Age** | | | | | | **Criteria** | | | | | | | | | | | | | | **Sensitivity** | | | | **PPV** | |
| 16-40 | | | | | | >10 cysts (Total) | | | | | | | | | | | | | | 100 | | | | 100 | |
| **ADPKD Exclusion** | | | | | | | | | | | | | | | | | | | | | | | | | |
| **Age** | | | | | **Criteria** | | | | | | | | | | | | | | | | **Specificity** | | | **NPV** | |
| >20 | | | | | <5 cysts (Total) | | | | | | | | | | | | | | | | 100 | | | 100 | |
| ADPKD: Autosomal dominant polycystic kidney disease, US: Ultrasound, CT: computed tomography, MRI: magnetic resonance imaging, PPV: positive predictive value, NPV: negative predictive value, FHx: family history | | | | | | | | | | | | | | | | | | | | | | | | | |

**Supplemental Table 2:** **Overview of the mechanism of action and the preclinical findings and clinical findings of emerging treatments for autosomal dominant polycystic kidney disease (ADPKD)**

| Categories | Mode of action | ADPKD preclinical studies | Clinical trials |
| --- | --- | --- | --- |
| **mTOR Inhibitors (3)**  Everolimus, Sirolimus | Inhibition of MDM2 prevents TP53 degradation and increases p21 expression à decrease cell proliferation and activation of apoptosis. | Induced disease regression by activating autophagy in ADPKD cells. | The sirolimus study (3) did not show a significant change in TKV or eGFR. however, the urinary albumin excretion rate was higher in the sirolimus group.  Everolimus slows TKV growth in patients with ADPKD but does not prevent kidney impairment progression. |
| **CFTR Modulators**  VX-809  GLPG2737 | CFTR chloride channel is responsible for driving net fluid secretion into the cysts, promoting cyst growth. Studies show that it is regulated by AVP (4). | An animal model of slowly progressing cyst formation typical of human ADPKD that VX-809 reduces the growth of already established cysts (5). | For VX809 a phase 2, double-blind, placebo-controlled followed by 1-year open label phase but was Terminated early due to lack of efficacy (ClinicalTrials.gov ID NCT04578548).  Phase 2 trial to assess safety and tolerability of GLPG2737 in ADPKD patients at risk of fast progression. |
| **Biguanide Analogues** | Metformin  Inhibits the mTOR and CFTR pathways and activates AMPK (6). | Metformin has been associated with reduced kidney cyst growth in an ADPKD mouse mode (6).  Diminishes leukocyte infiltration and downregulates inflammatory markers and kidney injury markers as well as accumulation of ECM in Rat kidney tubular epithelial cell line NRK-52E thus decreasing fibrotic changes in ADPKD (7). | Phase 2 showed no significant impact on TKV growth rate or kidney function.  In the present analysis, metformin also showed a favorable trend, but it was not significant, possibly because of a small sample size. IMPEDE-PKD phase 3 study aims to include a larger cohort (6). |
| **miRNA Inhibitors** Anti-miR-17  Anti-miR-21 | miR-17 rewires cyst epithelial metabolism to enhance cyst proliferation | anti-miR-17 demonstrated cyst-reducing effects, but no overt toxicity, in a second 6-month, preclinical trial involving a slow cyst-growth mouse mode (8) | A phase 1, with anti-miR-21, clinical study showed some changes in polycystin 1 and -2 levels in urinary exosomes  Current study is completed (ClinicalTrials.gov ID NCT04536688) Next phase study of Anti-mR-17 is under design at this point. |
| **Tesevatinib (KD019)** | Multi-kinase inhibitor targets multiple abnormal signal transduction events in PKD. Also targets abnormal angiogenesis necessary for cyst growth (9) | In vivo pharmacological inhibition of multiple kinase cascades with tesevatinib reduced phosphorylation of key mediators of cystogenesis: EGFR, ErbB2, c-Src and KDR which resulted in reduction of kidney and biliary disease in both bpk and PCK mice models of ARPKD (9) | Phase 2 trial with frequent adverse events, including QT-prolongation.  Study aiming to measure changes in htTKV was stopped 2 years ago (9-10) |
| **Somatostatin Analogues** | Lanreotide, Octreotide  Bind to SSTRs inhibiting AC activity and reduce cAMP production by maintaining intracellular Ca2+ levels (11). | - | Showed conflicting results in clinical trials.  Aladin trials showed reduced TKV growth but no eGFR decline whereas DIPAK-1 trial showed no significant impact on eGFR decline (12 , 13)  Several clinical studies have shown that somatostatin analogs inhibit not only kidney cysts but also hepatic cyst growth (11). |
| **Glucosylceramide Synthase (GCS) Inhibitors**  Venglustat  AL01211 | Designed to reduce the production of glucosylceramide (GL-1) and thus is expected to substantially reduce formation of glucosylceramide-based glycosphingolipids (14). | Reduced cyst growth and preserved kidney function in animal studies. | Venglustat trial was stopped due to insufficient effectiveness (ClinicalTrials.gov ID NCT04908462).  AL01211 is currently undergoing phase 1 clinical study ClinicalTrials.gov ID (NCT03523728). |
| **Nrf2 Activators**  Bardoxolone  Obacunone | Obacunone is a potent antioxidant that activates Nrf2 leading to suppression of lipid peroxidation and reduces cell proliferation by downregulating mTOR and MAPK signaling pathway (15). | In vitro obacunone significantly inhibited cyst formation and expansion of MDCK cysts and embryonic kidney cysts in a dose dependent manner  In vivo there was a significant reduction in kidney cyst formation (15). | Unpublished PHOENIX study indicated kidney function improvement. FALCON trial aimed to assess safety and efficacy but was eventually terminated (Clinicaltrials.gov NCT03366337, NCT0391844). |
| **Statin Therapy** | Inhibits 3-  hydroxy-3-methyl-glutaryl coenzyme A reductase, reduces the farnesylation and activation of  RAS guanosine triphosphate (GTP)-binding proteins that are important in numerous cellular  functions, including the regulation of cellular proliferation (16). | Animal models of ADPKD have shown that statin treatment decreases cyst formation, preserves kidney blood flow and mitigates interstitial inflammation (17). | Trials examining pravastatin's effect on TKV and combined with sodium citrate are still ongoing (18 , 19). The results of the pilot trial in adults with ADPKD, (presented at ASN 2024), did now show significant change in eGFR by using statins. |
| **Pioglitazone** | Decreased CFTR synthesis phosphorylation of Gab-1 leading to the downregulation of signaling pathways responsible of cell cycle and proliferation (20). | A substantial number of preclinical studies have found pioglitazone to decrease the cystic burden and improve the kidney function in ADPKD (20). | Phase 1b study showed safety in non-diabetic ADPKD patients but no observed effects on TKV or kidney function (19). |
| **Probenecid** | Uric acid lowering agent  ENaC activity inhibition  Reduces sodium levels and fluid retention within the cyst while increasing ENaC current and attenuates cyst formation (21). | Slows disease progression in a murine model of ADPKD (21). | Promising preclinical trials awaiting clinical trials. |
| ADPKD: autosomal dominant polycystic kidney, mTOR: mammalian target of Rapamycin, CFTR: Cystic fibrosis transmembrane conductance regulator, MDM2: mouse double minute 2 homolog, TKV: Total kidney volume, AVP: arginine vasopressin, AMPK: AMP- activated protein kinase, ECM: Extracellular matrix, PKD: polycystic kidney disease, EGFR: epidermal growth factor receptor, KDR: kinase insert domain receptor, htTKV: height adjusted total kidney volume, SSTR: Somatostatin receptor, AC: adenylyl cyclase, eGFR: Estimated glomerular filtration rate, GCS: glucosylceramide synthase, MDCK: Madin-Darby canine kidney cells, ENaC: Epithelial sodium channels | | | |
