# Supplementary figures and images for: Autosomal dominant polycystic kidney disease: an overview of recent genetic and clinical advances
Source: Ren Fail. 2025 Apr 23;47(1):2492374. doi: 10.1080/0886022X.2025.2492374 (PMC12020221; doi:10.1080/0886022X.2025.2492374)

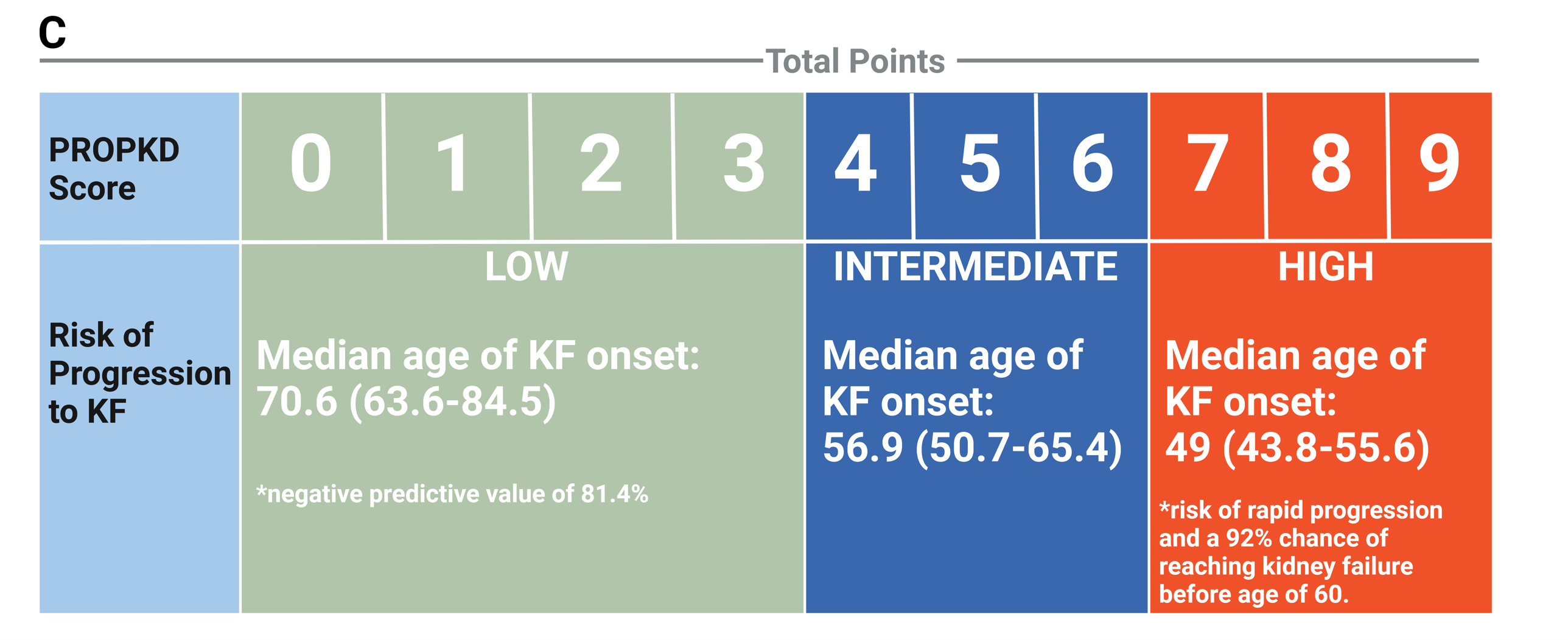

Supplement: SupFig7C.jpg [file IRNF_A_2492374_SM9116.jpg]

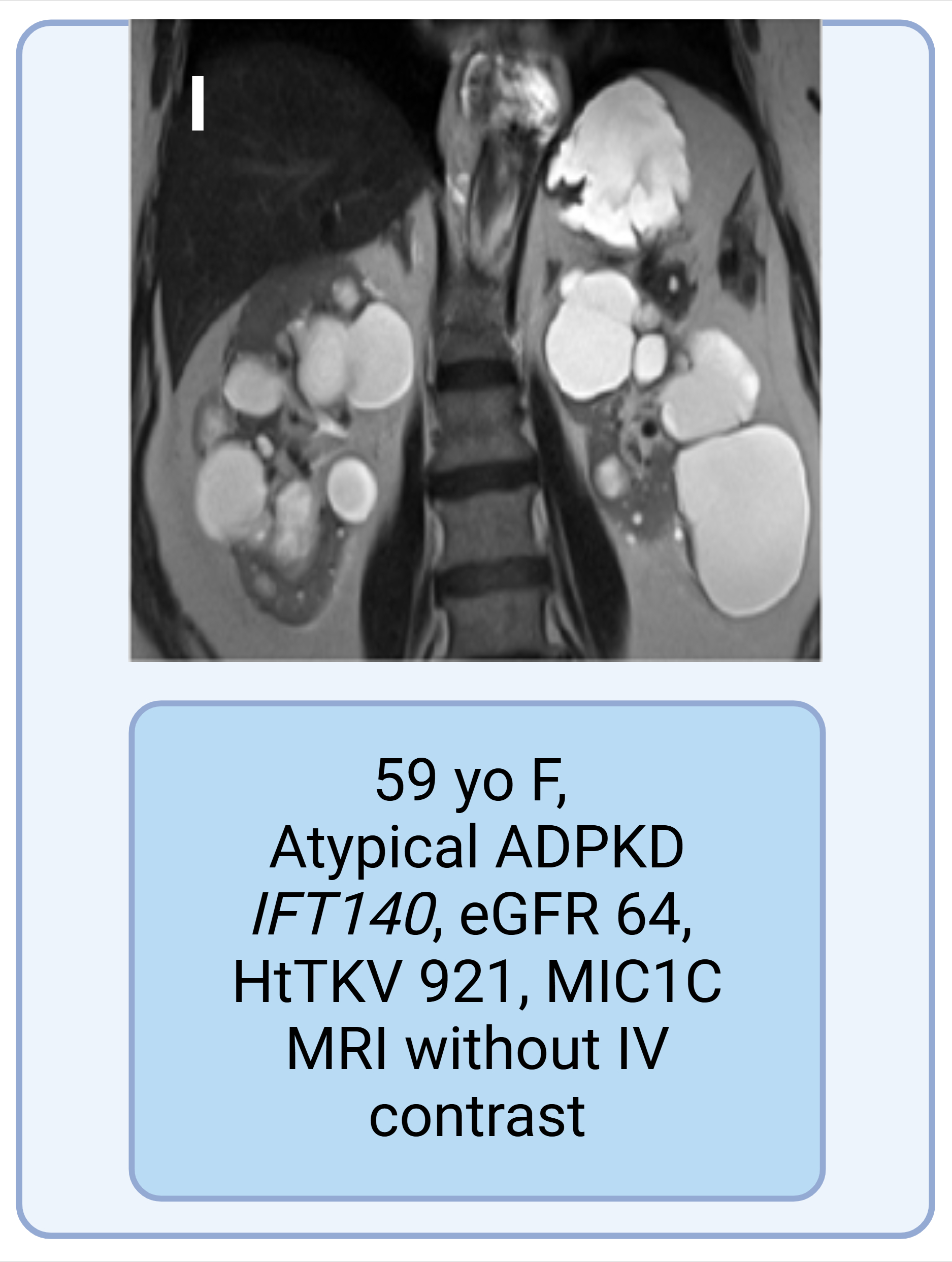

Supplement: SupFig 1i.jpeg [file IRNF_A_2492374_SM9099.jpeg]
